# Work-related problems faced by coordinators of organ, cell, and tissue transplantations in Poland and possible ways of ameliorating them

**DOI:** 10.1007/s10561-021-09982-0

**Published:** 2021-11-13

**Authors:** Artur Kamiński, Marcin Bury, Hanna Rozenek, Jolanta Banasiewicz, Stanisław Wójtowicz, Krzysztof Owczarek

**Affiliations:** 1 Department of Transplantology and Central Tissue Bank, Medical University of Warsaw, Chalubinskiego 5, 02-004 Warszawa, Poland; 2 National Centre for Cell and Tissue Banking, Chalubinskiego 5, 02-004 Warszawa, Poland; 3 Polish Transplant Coordinating Centre, al. Jerozolimskie 87, 02-001 Warsaw, Poland; 4 Department of Psychology and Medical Communication, Medical University of Warsaw, Zwirki i Wigury 81, 02-091 Warszawa, Poland

**Keywords:** Transplant coordinators; cell transplants, Tissue transplants, Tissue donors, Presumed consent

## Abstract

In recent years in Poland, the numbers of reported potential cadaveric donors of organs, tissues, and cells, and the numbers of transplantations being carried out seem to be low in the context of the size of the country population and the *presumed consent* legal principle which rules transplantations. This research project was carried out on 109 Polish transplant coordinators by means of a questionnaire created specifically for this study. The goal of the project was to detect problems specific to transplant coordinators working in Poland which, when properly addressed, might improve the efficacy of transplantation network within the Polish health care system. The results suggest that Polish transplant coordinators face a variety of issues in their work. It appears that the most important interventions which could improve working conditions for in this population and—as a result—also improve the efficacy of transplantation network in Poland could include: (1) a variety of training programs for transplant coordinators; (2) a social campaign promoting transplantations and spreading awareness of the transplantation-related legislation; and (3) introduction of changes in the regulations pertaining to medical professions in Poland.

## Introduction

The numbers of organ and tissue donors and organ and tissue transplantations in Poland were gradually increasing from the 1960s until 2005. After a decrease of several years’ duration, the numbers of organ transplantations increased again, but from 2014 to 2019 both the numbers of donors as well as organ and tissue transplantations have remained relatively at the same level in spite of high number of patients awaiting transplantation. The numbers of donors used for organ procurement oscillated between 504 and 594 per year, and the numbers of organs transplanted oscillated between 1446 and 1588 per year and between 12,000 and 13,000 tissues (from both organ and tissue and tissues donors) during that period. In the same period organ donors served as tissue donors between 400 and 450 per year while deceased tissue only donors oscillated between 700 and 800 per year. In 2019, 639 potential cadaveric organ and tissue donors were reported to the Polish Transplant Coordinating Centre, but only 79% of them were used for organ procurement. Out of those organ donors, 362 individuals were also tissue donors (tissues procured included the cornea, heart valves, musculoskeletal tissues, blood vessels and skin) (POLTRANSPLANT 2020).

In 2019, there were 301 donation coordinators working in 248 Polish hospitals. There are 386 hospitals in Poland which could report potential donors, but in 2019 only 133 hospitals reported potential donors to the transplantation network. Apart from the hospital coordinators, there were 27 regional transplant coordinators, and also 10 central transplant coordinators who worked in the Polish Transplant Coordinating Centre in 2019. Those 301 transplant coordinators working in hospitals included 193 medical doctors, 104 registered nurses, and 4 individuals working in other professions (paramedic, midwife, psychologist, and medical technologist) (POLTRANSPLANT 2020).

Little has been known about factors influencing the efficacy of transplantation network in our country and specific issues faced by transplant coordinators. The number of reported potential donors is definitely very small for a country of about 38 million inhabitants (Statistics Poland 2019) as it is believed that 2% of patients who died in hospitals and 15% of patients who died in intensive care units could become organ, tissue, and cell donors (POLTRANSPLANT 1998). A few years ago, coordinators from hospitals with small numbers of reported potential donors identified lack of support of their work as coordinators from co-workers and supervisors, who were not involved in the transplantation process, as the most important factor interfering with organ donations in Poland (POLTRANSPLANT 2017). Low social support at workplace from co-workers and supervisors was found to be negatively correlated with some aspects of professional burnout in this population (Bury et al. 2019). Another factor directly influencing work of transplant coordinators is inadequate knowledge by Polish society of the legislation pertaining to transplantation (the *presumed consent* principle) (CBOS 1997) and inadequate acceptance of transplantation as a treatment method (Ruszkowski et al. 2020). This issue seems not to be specific to Poland only as researchers from other countries have proposed commencing intense efforts to improve public awareness and knowledge about organ donation and transplantation in order to maximize donation and the overall success of transplantation (Siminoff et al. 2001; El-Shoubaki et al. 2006; Naghavi et al. 2020). In addition, it was proposed that relevant government agencies should focus on training nurses to discuss donation with bereaved families in order to improve donation rates (Naghavi et al. 2020).

This research project was intended to investigate work-related issues faced by a cohort of about a third of Polish transplant coordinators involved in organ, tissue, and cell transplantations. Due to lack of validated measures which could be used for this objective, we created a questionnaire specifically designed for the purpose of this study. Our goal was to detect problems specific to transplant coordinators working in Poland which, when properly addressed, could potentially improve the efficacy of transplantation network within the Polish health care system.

## Materials and methods

Participation in this anonymous study was completely voluntary. Participants received no compensation for taking part in this research project. The study measures were filled out by participants at a conference for transplant coordinators held in Warsaw, Poland.

The sample included a large cohort of hospital donation coordinators involved in organ, tissue, and cell transplantations, and it was the same cohort as the one used in our previous research project (Bury et al. 2019). In that past research project, we used several validated measures to investigate burnout and its correlates in Polish transplant coordinators. In the case the current study, however, we used a questionnaire created by us specifically for the purpose of this research. The research data were collected by means of questions answered on a five-point Likert scale (see the Tables—different questions had different answers format). In addition, we asked the participants to choose at least 5 issues—out of a list of 33 problems—which were most difficult to them in their work as transplant coordinators. The participants also had the opportunity to describe additional issues they faced at work, which were not included in that list. Finally, we asked the participants about their opinions regarding what form of contact with a psychologist would be most appropriate to meet their needs in their work as transplant coordinators (they were asked to check all the possible forms of psychological intervention they would be interested in on a 6-item list, and they could also give their own suggestions).

## Results

### General characteristic of the study sample

The study sample consisted of 109 participants, i.e., transplant coordinators involved in organ, tissue, and cell transplantations in Poland. This group included 36 men (33%) and 73 women (67%), with mean age of 45.32 years (SD = 8.41). Their average time of working as transplant coordinators was 7.73 years (SD = 5.26).

In Poland, there is an administrative/legal distinction between a village (which is a small, rural entity without legal town privileges) and a town (which is bigger and does have legal town privileges). Only one participant (0.9%) of our study worked in a village area, 18 (16.5%) worked in a city with a population below 50,000 inhabitants, 31 (28.4%)—in a city with a population between 50,000 and 200,000 inhabitants, 20 (18.3%)—in a city with a population between 200,000 and 500,000 inhabitants, and 39 individuals (35.8%) worked in the largest Polish cities, which populations exceeded 0.5 million inhabitants. Sixty-seven percent of participants were in a relationship while 33% declared to be single. Ninety-seven participants (89.8%) reported to be religious, with different degree of participation in religious practices (from regular participation to no participation). Fifty individuals (45.9%) reported that within the previous year they had experienced no difficult situations in their personal life, 32 (29.4%) reported that such situations had happened once, 26 (23.9%) reported that such situations had happened 2–3 times, and one individual (0.9%) reported that they had experienced such situations 4–5 times within the previous year.

Over 80% of participants described the support they were receiving from their families as Good or Very Good (see Table 1). Similarly, over 72% of the sample described their financial situation as Good or Very Good (Table 1).

**Table 1:** Results for questions

	Q1		Q2	
	N	%	N	%
Very bad	1	0.9	1	0.9
Bad	1	0.9	1	0.9
Average	19	17.4	28	25.7
Good	42	38.5	58	53.2
Very good	46	42.2	21	19.3

N—number of individuals who have endorsed the answer; %—percent of the total sample

Q1: How would you describe the support that you have been receiving from your family?

Q2: How would you describe your current financial situation?

### Reported influence of work on participants

Slightly over a third of the sample (36.2%) admitted to be Frequently or Always thinking about the donors they were dealing with while working as transplant coordinators, while the remaining participants were thinking about them Sometimes, Rarely, or Never (Table 2). Slightly over 70% of individuals disclosed Rarely or Never thinking about a person as a collection of cells or organs, while the remaining participants admitted having such thoughts Sometimes or Frequently (Table 2). Over 75% of participants reported to Never or Rarely feel alienated in social situations because of their experiences while working as transplant coordinators (Table 2). Close to 40% of participants admitted to Frequently or Always think that working as a transplant coordinator was emotionally exhausting, and over 51% reported that their family and friends described their job as very difficult (Table 2).

**Table 2:** Results for questions

	Q3		Q4		Q5		Q6		Q7	
	N	%	N	%	N	%	N	%	N	%
Never	6	5.6	47	43.1	46	42.2	5	4.6	2	1.8
Rarely	16	14.8	30	27.5	36	33.0	12	11.0	12	11.0
Sometimes	47	43.5	20	18.3	23	21.1	49	45.0	39	35.8
Frequently	33	30.6	12	11.0	3	2.8	33	30.3	49	45.0
Always	6	5.6	0	0	1	0.9	10	9.2	7	6.4

N—number of individuals who have endorsed the answer; %—percent of the total sample

Q3: How often do you think about the donors whom you deal with while working as a transplant coordinator?

Q4: How often do you think about a person as a collection of cells or organs?

Q5: How often do you feel alienated in social situations because of your experiences while working as a transplant coordinator?

Q6: How often do you think that working as a transplant coordinator is emotionally exhausting?

Q7: How often do the people you interact with (your family, friends) describe your job as very difficult?

### Reported need for psychological services

Over 44% of transplant coordinators who took part in our study reported that Definitely or Probably they did not need access to professional psychological services because of the nature of their work, while almost a quarter (24.5%) had no opinion (Table 3).

**Table 3:** Results for the question:* Do you think that, because of the nature of your work as a transplant coordinator, you need access to professional psychological support?*

	N	%
Definitely not	13	12.3
Probably not	34	32.1
It’s hard to say	26	24.5
Probably yes	25	23.6
Definitely yes	8	7.5

N—number of individuals who have endorsed the answer; %—percent of the total sample

When asked what kind of support they would find most appropriate to meet their needs, suppose they were able to have contact with a psychologist at work, almost half of the participants (47.7%) endorsed trainings (e.g., how to converse with the families of potential donors; how to cope with stress), while over a third (35.8%) marked individual sessions with a psychologist (individual psychological sessions are aimed at solving psychological problems of the client). Thirty-four percent reported interest in participating in various support groups (Table 4). Out of the 9 individuals (8.3%) who chose “Other” as an option, only 3 described a form of psychological intervention while 3 requested help with solving non-psychological problems at work, and 3 stated they did not want to participate in any contact with a psychologist.

**Table 4:** Results for the question: *If you were able to have contact with a psychologist, what kind of support would you find most appropriate to your needs?*

	N	%
Individual sessions with a psychologist	39	35.8
Support group for transplant coordinators only	21	19.3
Support group for people involved in the process of organ / cell transplantation	16	14.7
Training (e.g., how to converse with the families of potential donors; how to cope with stress)	52	47.7
Constant availability of psychological support at my work place	20	18.3
Other	9	8.3

The participants could mark more than one option. N—number of individuals who have endorsed the answer; %—percent of the total sample

### Reported work-related problems

Table 5 presents the frequency of endorsing options from a list of the most difficult aspects of work as a transplant coordinator. Among the 13 most-frequently-endorsed items (each was marked by over 20% of the participants), the following groups of issues could be distinguished: (1) problems related to psychological distress resulting from different aspects of interactions with families in the context of death of the potential donor; (2) issues resulting from lack of knowledge of transplantation laws and lack of societal understanding of using transplantation as a treatment method; and (3) complaints pertaining to unfavorable work conditions and negative social interactions at workplace (see Table 5 for details).

**Table 5:** The most difficult aspects of work as a transplant coordinator

	N	%
Direct contact with families of potential donors	55	50.5
Work conditions (lack of necessary equipment or space, logistic problems, etc.)	49	45.0
Lack of knowledge of the transplantation-related legislation by the society and donors’ families	44	40.4
Lack of understanding by the medical personnel at my work place of the negative emotions experienced by me in my work	42	38.5
Lack of understanding/acceptance by the society of using organ/tissue transplantation as a treatment method	37	33.9
Interpersonal conflicts at workplace	33	30.3
Daily contact with death and human tragedies	33	30.3
Lack of understanding by my supervisors at my work place of the negative emotions experienced by me in my work	31	28.4
My inability to have control over or to influence my work place	31	28.4
Lack of professional psychological support for transplant coordinators	30	27.5
Subjective sense of loneliness resulting from lack of help/cooperation with me by medical personnel at my work place	29	26.6
Lack of support to transplant coordinators from the institution I am employed at	27	248
Subjective sense of loneliness resulting from lack of help/cooperation with me by my supervisors at my work place	22	20.2
Lack of time for satisfactory private life because of the amount of time I devote to my work as a transplant coordinator	19	17.4
Subjective feeling of emotional exhaustion	19	17.4
Being unable to participate in the decision making process at my work place	19	17.4
Phone conversations with the families of potential donors about donation	18	16.5
Lack of cooperation by the medical personnel whom I contact while carrying out my duties of a transplant coordinator	17	15.6
Lack of clarity regarding my work duties of a transplant coordinator	16	14.7
Lack of physical/psychological energy	14	12.8
Too much professional responsibility related to working as a transplant coordinator	13	11.9
Stress, which forces me to use substances in order to relax (alcohol, cigarettes, medicines, illicit drugs)	11	10.1
Lack of understanding by my family and friends for the negative emotions experienced by me at work	11	10.1
Subjective feeling of being overwhelmed by my work as a transplant coordinator	10	9.2
Lack of knowledge about the results of my work (i.e., lack of knowledge about the fate of transplant recipients)	10	9.2
Subjective feeling of achieving no success at my work	10	9.2
Excessive professional requirements for transplant coordinators	9	8.3
Participating on organ/tissue harvesting	7	6.4
Lack of satisfaction from working as a transplant coordinator	5	4.6
The view of dead human bodies	4	3.6
Subjective feeling that there is no sense in my work as a transplant coordinator	2	18
The view of harvested fragments of human bodies	2	18
The place in which organ/tissue harvesting takes place	1	0.9

The participants were asked to choose at least 5 answers. N—number of individuals who have endorsed the answer; %—percent of the total sample

The participants were also given an opportunity to describe other most difficult aspects of their work as transplant coordinators, which were not included in the list presented in Table 5. Thirty-two individuals reported that for them the most difficult aspects of their jobs included also: different forms of interpersonal conflicts (15 participants), lack of time (6 participants), psychological stress caused by factors other than contacts with donors’ families (e.g., problems with obtaining financial reimbursement for work) (5 participants), not enough donors (2 participants), contacts with donors’ families (2 participants), lack of personnel (1 participant), and not enough paid leave time (1 individual).

## Discussion

Our sample consisted of organ, tissue and cell transplant coordinators who, on average, had several years of work experience and most of whom worked in larger cities, which roughly reflected the structure of transplantation network in Poland. Because this study included only about 36% of all Polish hospital donation coordinators, its results did not reflect the situation of this entire vocational group, and more research carried out on higher number of participants may be needed to obtain a more reliable assessment of problems that Polish transplant coordinators face at their work.

In comparison with the general population in our country, the percentage of participants who were in a relationship was higher than that in the general population (Statistics Poland 2019), which was probably related to the age structure of our study cohort. Most of them positively described the support they received from their families, and many of them reported than their family members and friends perceived their job as very difficult. Only every tenth study subject complained of lack of understanding by his families and friends to the negative emotions they experienced at work, which is important as the quality of social support has been found to be negatively correlated with emotional exhaustion and reduced personal accomplishment (Mao et al. 2018). All these factors might be suggestive of better assistance from family members and friends than may be the case for an average member of Polish society. Similarly, the study cohort’s subjectively-perceived financial situation was better than that of the general population in Poland (CBOS 2016), while their religious affiliation level seemed to mirror that of the Polish society (European Commission EURYDICE 2021). Three-quarters of participants reported to have experienced none or only one difficult situation in personal life within the previous year. In summary, it might seem that the participants of our research project enjoyed somewhat better life circumstances in comparison with random representatives of the Polish nation. Unfortunately, the specific work conditions related to coordinating cadaveric donations within the Polish health care system (conditions which contribute to psychological distress; Bury et al. 2019) make this picture not so bright.

Only less than a third of our sample believed they needed access to professional psychological services because of the nature of their work as organ, tissue, and cell transplant coordinators. At the same time, however, half of them disclosed that the most difficult aspect of their work was direct contact with the families of potential donors, while almost every third complained of daily contact with death and human tragedies, and every sixth endorsed phone conversations with the families of potential donors about donation. Because only very few of the transplant coordinators reported issues with having to participate in harvesting of organs, tissues, and cells, or seeing dead human bodies or their fragments, it appears that contacts with grieving members of donor families, which can give raise to significant psychological distress (Luo et al. 2020; Silva e Silva et al. 2020), are the most difficult work-related issue in this population. This is reflected by the finding that almost half of the subjects in our study pointed out to training (e.g., how to converse with the families of potential donors about donation, or how to cope with stress) as the most useful form of psychological support in their work. It appears that in our country coordinators of organ, tissue, and cell transplantations would benefit from a variety of training events carried out by psychologists, which could be delivered, e.g., during transplantation-focused conferences. Such training programs could address communication skills necessary for interacting with grieving donor families, ways of improving coordinators’ psychological resilience, or promoting psychological self-care and preventing work-related burnout among transplant coordinators.

It should be noted, however, that in spite of all the above issues and the fact that close to 40% of our sample found their job emotionally exhausting, our study cohort seemed to be doing quite well in terms of preoccupation with the donors they deal with, becoming insensitive and thinking about a person as a collection of cells or organs, or feeling alienated in social situations because of their job-related experiences.

Another difficult aspect of work as organ, tissue, and cell transplant coordinator in Poland identified by our study was lack of knowledge by the society of Polish legal regulations pertaining to transplantation (including families of potential donors) and lack of societal understanding of using transplantation as a treatment method. In Poland the *presumed consent* legal principle is used, and every citizen over 16 years of age is regarded as a potential donor unless that person officially declares their objection to donate their organs, tissues, or cells, which can be done by officially informing the Polish Transplant Coordinating Centre, or carrying at all times a signed objection letter, or verbally informing at least two witnesses (they would need to confirm this verbal objection in writing in case of death of the person making the objection). In the case of a person under 16, during their life the objection can be made by their legal guardian (Polish Transplant Coordinating Centre official webpage xxxx). Such *presumed consent* legislation was found to have positive effects on cadaveric organ donation rates (Abadie and Gay. 2006; Shepherd et al. 2014). However, in a study carried out 20 years before our research project (CBOS 1997), only 18% of Polish respondents knew about those regulations, and based on our results it can be suspected that until now the social awareness of the legislation remains limited, even though the number of objections sent to the Polish Transplant Coordinating Centre has been increasing since 2014 (Kozlowski 2018). It is justifiable to suspect that lack of knowledge about Polish transplantation-related legislation among families of potential donors makes the work of transplant coordinators much harder in our country. We don’t know the current situation, but in the 1990s 64% of surveyed adult Poles believed that before harvesting organs for transplantation medical doctors ought to ask the deceased person’s family members if they were against the organ donation or not (CBOS 1997). Such a stance can make donation-related conversations especially difficult for transplant coordinators.

When it comes to societal acceptance for transplantation as a treatment method, in a recent study (Ruszkowski et al. 2020) only 64.2% of respondents reported they believed that it was *definitely* right to use organs from cadaveric donations to save life or restore health in transplant recipients. It appears, however, that younger Poles have more favorable approach towards transplantation as in a different study 95.7% of surveyed high school and university students declared their acceptation for transplantation as a treatment method (Wojciechowski et al. 2012). Nonetheless, the numbers of organ transplantations in Poland have remained more-or-less stable in the recent years, and the steady increase in transplantation numbers observed earlier (e.g., until 2005) has ceased, which is worrisome considering high numbers of recipients awaiting transplantations (POLTRANSPLANT 2020).

Overall, it appears that a nation-wide campaign in our country, focused on educating on transplantation-related laws and promoting organ, tissue, and cell transplantations as a valid, ethical treatment modality could contribute to decreasing work-related stress among Polish transplant coordinators and increase the efficacy of transplantation network.

In Poland, transplant coordinators work as coordinators on top of their primary job (which could be, e.g., a nurse or a medical doctor). Therefore, it comes as no surprise that, among the remaining most difficult aspects of work pointed out by the participants of our study, the most endorsed ones pertain to negative work conditions and lack of support/understanding/help/cooperation from people they work with, including their supervisors and medical personnel. This cluster of problems can exert negative influence on cadaveric transplantations efficacy in our country in several ways. In a survey of Polish transplant coordinators (POLTRANSPLANT 2017), coordinators from hospitals with small numbers of reported potential donors identified lack of support from co-workers and supervisors as the most important factor interfering with organ donations. In addition, support from supervisors can help transplant coordinators solve problems and reduce work-related stress (Luo et al. 2020), and social support was found to be negatively-related to emotional exhaustion and reduced personal accomplishment in this population (Mao et al. 2018). Negative correlations between burnout and social support from supervisors have been reported in the populations of employees of cell and tissue banks (Kaminski et al. 2018) and transplant coordinators (Bury et al. 2019). It can be suspected that improving cooperation between transplant coordinators and the personnel of hospitals they work in, including their supervisors, could result not only in decreased work-related stress in coordinators but also in greater efficacy of transplantation network in our country. This, however, would require broad, systemic adjustments in legislation ruling medical professions, including recognition of transplant coordinator as a profession, giving coordinators more privileges, and forcing hospital stuff to give precedence to coordinators’ requests because of the time constraints related to organ, tissue, and cell harvesting. This would also require a change in the mentality of hospital personnel, which may be even harder to achieve than legal changes.

The results of this research project suggest that Polish transplant coordinators face a variety of issues in their work. It appears that the most useful interventions which could improve the quality of work in this population, and—as a result—also the efficacy of transplantation network in our country, include training programs for transplant coordinators, a social campaign promoting transplantations and spreading awareness of the transplantation-related laws, and adjustments in the legislation pertaining to medical professions in Poland.
